# Aspartic Acid Stabilized Iron Oxide Nanoparticles for Biomedical Applications

**DOI:** 10.3390/nano12071151

**Published:** 2022-03-30

**Authors:** Mihaela Răcuciu, Lucian Barbu-Tudoran, Simona Oancea, Olga Drăghici, Cezarina Morosanu, Marian Grigoras, Florin Brînză, Dorina E. Creangă

**Affiliations:** 1 Environmental Sciences and Physics Department, Faculty of Sciences, “Lucian Blaga” University of Sibiu, Dr. I. Ratiu Str., no. 5-7, 550012 Sibiu, Romania; 2 Electron Microscopy Integrated Laboratory, National Institute for R&D of Isotopic and Molecular Technologies, Donat Str., no. 67-103, 400293 Cluj-Napoca, Romania; lucian.barbu@itim-cj.ro; 3 Electron Microscopy Laboratory “Prof. C. Craciun”, Faculty of Biology and Geology, Babes-Bolyai University, Clinicilor Str., no. 5-7, 400006 Cluj-Napoca, Romania; 4 Agricultural Sciences and Food Engineering Department, Lucian Blaga University of Sibiu, Dr. I. Ratiu Str., no. 7-9, 550012 Sibiu, Romania; simona.oancea@ulbsibiu.ro (S.O.); olga.draghici@ulbsibiu.ro (O.D.); 5 Biophysics and Medical Physics Laboratory, Faculty of Physics, “Alexandru Ioan Cuza” University, 11, Carol I Blvd., 700506 Iasi, Romania; cezarina-morosanu@yahoo.com (C.M.); fbrinza@uaic.ro (F.B.); mdor@uaic.ro (D.E.C.); 6 National Institute of Research and Development for Technical Physics, 47, Mangeron Blvd., 700050 Iasi, Romania; mgrigoras@phys-iasi.ro

**Keywords:** iron oxide nanoparticles, nanoparticles synthesis, L-aspartic acid, nanoparticles size, physical and chemical characterization

## Abstract

Aspartic acid stabilized iron oxide nanoparticles (A-IONPs) with globular shape and narrow size distribution were prepared by the co-precipitation method in aqueous medium. A quantum-mechanical approach to aspartic acid optimized structure displayed negative charged sites, relatively high dipole moment, and hydrophilicity, which recommended it for interaction with iron cations and surrounding water electrical dipoles. A-IONPs were characterized by TEM, XRD, ATR-FTIR, EDS, DSC, TG, DLS, NTA, and VSM techniques. Theoretical study carried out by applying Hartree-Fock and density functional algorithms suggested that some aspartic acid properties related to the interaction can develop with nanoparticles and water molecules. The results of experimental investigation showed that the mean value of particle physical diameters was 9.17 ± 2.2 nm according to TEM image analysis, the crystallite size was about 8.9 nm according to XRD data, while the magnetic diameter was about 8.8 nm, as was determined from VSM data interpretation with Langevin’s theory. The A-IONP suspension was characterized by zeta-potential of about −11.7 mV, while the NTA investigation revealed a hydrodynamic diameter of 153.9 nm. These results recommend the A-IONP suspension for biomedical applications.

## 1. Introduction

Nanotechnology development has provided a wide range of nanomaterial designs that are useful and necessary in modern people’s lives, with applications in both technical and biomedical fields. In the sanitary domain, gelatin-based nanofiber dressings were found to be very useful for wound healing applications [1]; in biomedicine, therapeutic nanoparticles loaded with pharmaceutical agents have been used, such as, for example, gel beads and fibers for co-delivery of multiple agents, [2] or super molecule nanoparticles for tumor suppression and synergistic immunotherapy [3], and exosome-nanoparticles for treating brain disorders [4]. However, our knowledge about the interactions of nanomaterials with living cells hasn’t kept up with this development and is still not understood well enough. Magnetic nanoparticles have aroused great interest. Biocompatible iron oxide nanoparticles suspensions have biological and medical applications on an extensive scale, such as in the medical diagnostic field and in therapy [5,6,7,8]. The stability and biocompatibility of iron oxide nanoparticles’ colloidal suspensions are affected by their particle size distribution as well as by the nature of the surface coating of the nanoparticles in the solution [9]. Iron oxide nanoparticles are usually stabilized and functionalized with a large variety of organic compounds from small molecules to polymers to make them suitable for various purposes [10]. The biomedical applications of magnetic nanoparticles demand a peer control of the interactions with cells and biological fluids that are strongly related to surface layer properties [11]. Thus, the compounds modifying the magnetic nanoparticle surface should be nontoxic and biocompatible. A suitable surfactant ensures nanoparticle stability in suspension, by being able to generate the repulsive forces between particles in order to prevent aggregations [12]. Iron oxide nanoparticles have attracted much interest as contrast agents in magnetic resonance imaging, as drug carriers, and for magnetic hyperthermia in cancer therapy. The best of these nanoparticles are designed to combine small size, biocompatibility, heating ability, cost, and versatile synthesis. For biomedical applications, iron oxide nanoparticles with a physical diameter of about 5–20 nm seem to be the ideal candidate [13]. Most often, the co-precipitation method for the iron oxide nanoparticle synthesis is considered one of the simplest, most available, and cheapest methods. Recently different amino acids were found to be promising coatings for biocompatible nanoparticles: adequate for preparation of iron-oxide nanoparticle suspensions with no particle aggregations [14,15,16,17,18]. In addition, biological applications have been developed; natural amino acids have been recognized as an excellent option to coat iron oxide nanoparticles used in food processing [19,20], while arginine, lysine, and polylysine-coated magnetic nanoparticles were prepared for applications in bacterial capture [21]. Aspartic acid containing amino and carboxylic groups, known to be involved in protein biosynthesis, was recently found to have an important role in the immunoglobulin production and antibody synthesis [22]. Generally, the L-aspartic acid is also considered as a secondary excitatory neurotransmitter [23]. Aspartic acid-functionalized nanoparticles for biomedical use are presented by Singh et al. (2016) [24], and Ebrahinimezhad et al. (2012) [25,26], and are discussed in Nosrati et al. (2017) [27] by comparison to other molecule-stabilized nanoparticles. Other authors, including Afradi et al. (2019), studied aspartic-acid-loaded starch functionalized ferrite magnetic nanoparticles as catalysts [28]. Regarding the interaction mechanisms, Nosrati et al., the authors of [27], sustained the aspartic acid binding with MNP surface through carboxyl groups.

This study has been designed for the synthesis of iron oxide nanoparticles stabilized with aspartic acid (A-IONPs) by the co-precipitation method and the study of their properties. Comparison of some experimental data and theoretically computed ones was carried out with a focus on the characteristics and peculiarities of the A-IONPs’ molecular surface modifier: aspartic acid.

## 2. Materials and Methods

Materials. Ferrous chloride tetra-hydrate (FeCl_2_·H_2_O), ferric chloride hexa-hydrate (FeCl_3_·6H_2_O), L-aspartic acid (C_4_H_7_NO_4_), and 25% ammonium hydroxide (NH_4_OH) (J.T. Baker) were used, according to the synthesis protocol from [29]. Distilled water used in all experimental procedures was obtained by means of a water-still (DEST3—BOECO—Germany).

### 2.1. Yielding of A-IONP Aqueous Suspensions

An A-IONP synthesis protocol, based on an adapted approach of controlled chemical precipitation at room temperature [29] presented in [30] was applied for core/shell nanosystem yields. In practical terms, two acidic solutions of ferric and ferrous salts were prepared in 2 M HCl solution: 8.0 mL of 2 M stock FeCl_2_ solution and 32.0 mL of 1 M stock FeCl_3_ solution. The two precursor solutions were magnetically mixed at room temperature with 400 mL of 1 M aqueous NH_4_OH solution that was poured at a constant flow rate of 2 mL per second, as precipitation agent. Immediately afterwards, stirring was stopped and the resulted precipitate was magnetically isolated using a strong permanent magnet placed under the reaction vessel. The supernatant was removed and then the synthesized ferrophase was washed several times with a total volume of about 1–1.5 L of distilled water to remove mainly the ammonium chloride resulted from the precipitation process. The obtained dark-brown precipitate (100 mL volume of wet ferrophase) was mixed with 0.8 g aspartic acid (C_4_H_7_NO_4_) diluted in 50 mL distilled water, and then vigorously mixed for 150 min—with 500 rpm mechanical stirrer. 

### 2.2. Characterization of A-IONPs 

The morphology and diameter of the nanoparticles were estimated from TEM imaging captured from dried samples deposited on 400 mesh carbon-coated copper grids. Images were taken using a Hitachi HD2700 CFEG STEM, at 200 kV with secondary electron imaging capability. EDS investigation was also accomplished, obtaining information about the elemental composition of the nanoparticle sample. XRD analysis—Crystalline features were studied by means of X-ray diffraction pattern (XRD) recorded by using a Shimadzu 6000 XRD device with Cu–Kα radiation at λ = 0.15418 nm between 20 and 80 degrees (I = 25 mA, U = 35 kV, 0.004 degree/step, 1.2 s measuring time per step) in a Bragg–Brentano arrangement. The ATR-FTIR spectrum was acquired using an ATR-FTIR Alpha spectrometer (Bruker, Germany). The ATR-FTIR spectrometer works with a ZnSe crystal; it is a non-destructive and preferred method for fluid sample analysis. All the ATR-FTIR spectra recordings were conducted at room temperature, at about 21 °C. Resolution was of 4 cm^−1^ with the spectral range of 4000–600 cm^−1^. DSC and TG analyses were performed between 25 °C and 850 °C, at the heating rate of 10 °C/min, using the SDT Q600 calorimeter (TA Instruments). The A-IONP sample scanning was performed in nitrogen atmosphere at a flow rate of 50 mL/min. The sample mass was about 15 mg, and ceramic pans were used. The heat flow data, registered by DSC, are given in W/g, while the weight loss data, registered by TG are given in percentage. The Universal Analysis 2000 software, supplied by TA Instruments, was used for data analysis. The study of the magnetization curve with focus on the saturation magnetization and coercive field of A-IONPs was accomplished by the vibrating sample magnetometry method (VSM). MicroMag Model 2900/3900 magnetometer was used at a room temperature of about 24.0 ± 0.5 °C. For the 10^−3^ volume diluted sample, the electric conductivity was measured by means of digital WTW Multi 3430 -F meter (Germany). For the estimation of the zeta-potential of nanoparticle suspension nanoparticles by dynamic light-scattering method (DLS), a Malvern Zetasizer Nano ZS, model Zen-3500, was used at room temperature of about 24.0 ± 0.5 °C. To obtain the nanoparticle size distribution in liquid suspension, the nanoparticle tracking analysis (NTA) was applied, to estimate the hydrodynamic diameter of the magnetic cores using a NanoSight LM20 device (at room temperature of about 24.0 ± 0.5 °C). For DLS and NTA measurements, we worked with 10^−4^ volume dilution of the A-IONP sample.

### 2.3. Theoretical Approach of Molecule Structure and Properties

The mathematical modeling of the coating molecule—the aspartic acid—was carried out by means of specialized software Spartan’14 to get the optimized structure of the amino acid, some of its essential characteristics, and its optical spectra, based on two mathematical approaches, i.e., Hartree-Fock 3-21 G* and Density-Functional EDF2, 6-31 G* [31,32].

## 3. Results

**Figure 1:**
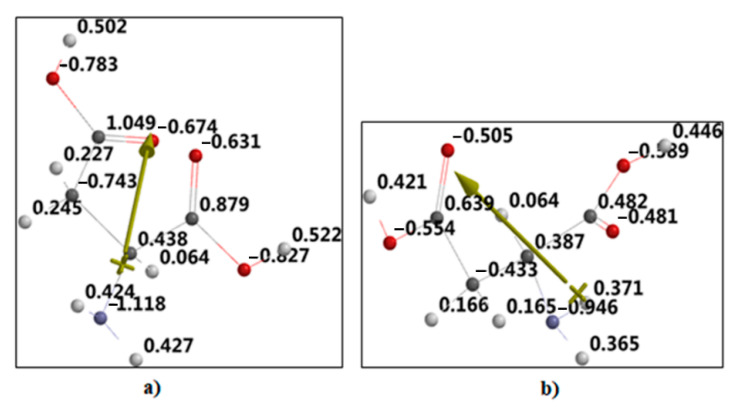
Electric charge distribution corresponding to optimized molecular structure in the two quantum-chemical approaches: (**a**) Hartree-Fock, (**b**) Density-Functional methods. Arrow represents the direction of the electrical dipole moment.

A theoretical approach to aspartic acid properties—enabling it to interact with magnetic nanoparticles and environmental aqueous media—was carried out. The calculated electric charges corresponding to different atoms are presented in Figure 1.

For example, the negative charge on the nitrogen atom is over the unit (−1.18e, Figure 1a), versus sub unitary value (−0.948e, Figure 1b)—thus the rising dipole moment from nitrogen atoms being oriented toward oxygen ones. The carboxylic edges have oxygen atoms with more than −0.6e charges: −0.783e, −0.674e, −0.631e, and −0.827e in the first modeling (Figure 1a); while in the second case (Figure 1b) the values are under −0.6e: −0.505e, −0.554e, −0.59e, and −0.481e), where “e” is the elementary electric charge.

**Figure 2:**
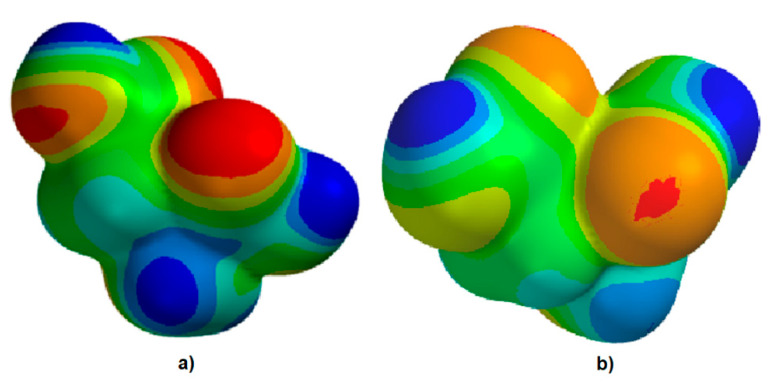
Electrostatic potential map generated with Hartree-Fock (**a**) and Density Functional (**b**) methods.

In both mathematical approaches, electrostatic potential map representation (Figure 2) has shown higher values at the level of oxygen sites (reddish to red colored spots), where interactions with iron cations at the nanoparticle surface could be favored.

Calculated parameters have similar values in both approaches, such as solvation energy (−53.06 kJ/mol for Hartree Fock modeling and −55.27 kJ/mol for DFT modeling), polarizability (47.34 Å^3^ and 49.39 Å^3^), and polarized surface area (91.964 Å^2^ and 92.922 Å^2^, respectively).

In addition, remarkable dipole moment values (1.6 D versus 0.91 D)—lower but comparable with that of water molecules (1.8 D)—suggest the possible development of strong interactions with iron cations, fulfilling the basic condition of magnetic core coating with aspartic acid molecules in accordance with Sapic et al. (2019), who also reported 1.6 D dipole moment calculated by DFT simulation under Gaussian soft package [33]. Other authors reported an aspartic acid dipole moment of around 4 D [34].

Theoretical modeling let us anticipate not only that aspartic acid could develop good interactions with iron cations at the nanoparticle surface but also ensured that the nanoparticles were floating in aqueous media.

**Figure 3:**
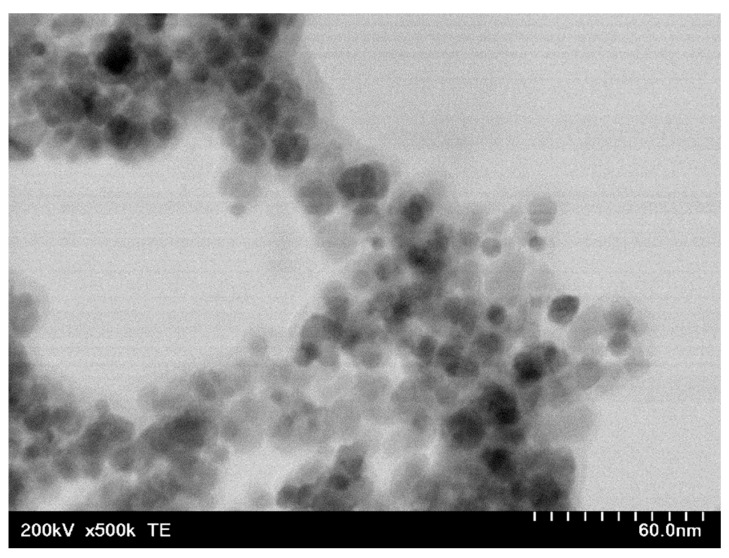
TEM image of iron-oxide nanoparticles (scale bar = 60 nm).

**Figure 4:**
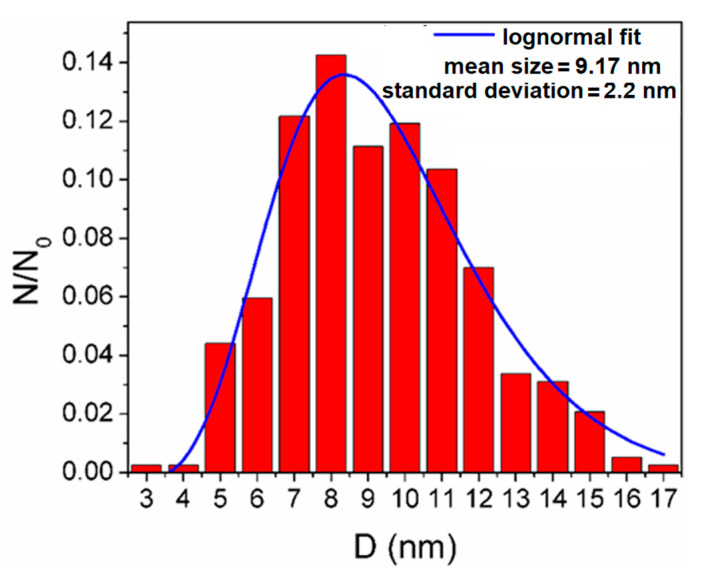
The size distribution of iron-oxide nanoparticles on 336 particles from 14 different TEM pictures.

Experimental results are presented next. The size distribution of A-IONPs resulted from the analysis of TEM images (Figure 3), and were fitted using a log-normal function, using OriginLab software (Figure 4). They showed the physical diameter of A-IONPs to be about 9.17 ± 2.2 nm (R- square value of 0.9491), using 336 particles from 14 different TEM pictures with Image J software. From a lognormal distribution fit, the median value of nanoparticle size was 9.17 nm with a standard deviation of about 2.2 nm.

As seen in the TEM images, the A-IONPs are well dispersed, having a globular shape with around 9 nm mean size and a rather narrow size distribution, mostly between 3 and 17 nm, without visible difference in the morphology. A low degree of the A-IONP agglomeration was revealed by means of detailed TEM image analysis. The narrow size distribution makes the nanoparticles suitable for using in the biomedical field. 

**Figure 5:**
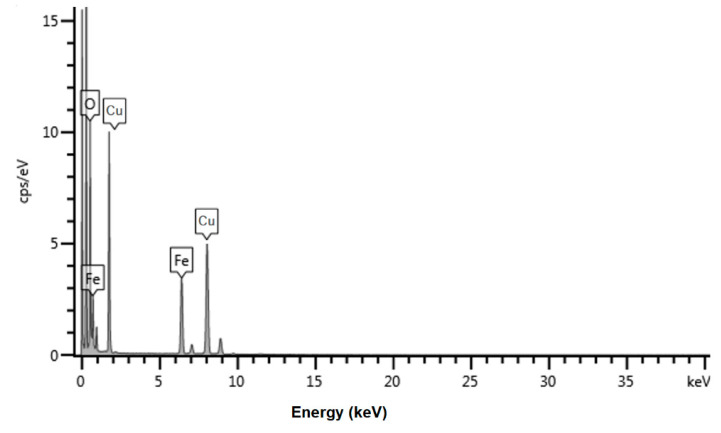
EDS pattern of the synthesized A-IONPs.

The elemental analysis of nanoparticles (EDS pattern) confirmed the presence of iron and oxygen (Figure 5). No other signal was identified, considering the EDS analysis threshold. The additional peaks correspond to copper from copper grid supports for the sample deposition during the TEM analysis.

**Figure 6:**
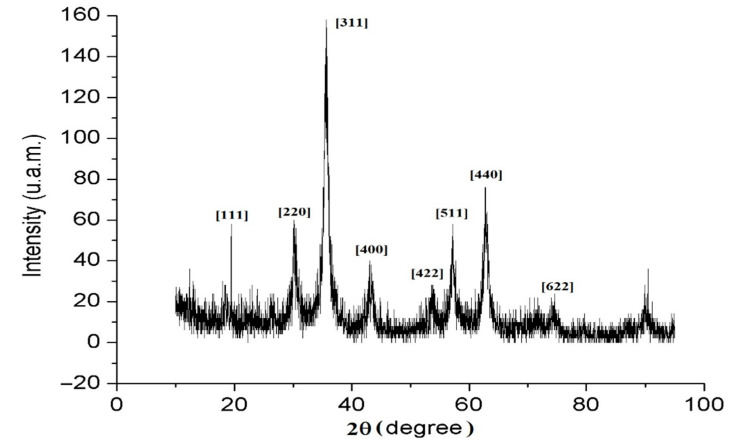
X-ray diffraction pattern of the synthesized A-IONPs.

Figure 6 shows the XRD pattern for an A-IONP sample. The crystallite size of nanoparticles sample was estimated using the Scherrer equation [35] applied to the highest peak (311):

(1) $$D_{\mathrm{hkl}}=\frac{k\cdot \lambda }{\beta \cdot \cos\theta }$$

where D_hkl_ (nm) is the size of crystallites, k is crystallite shape factor (with a good approximation at 0.9), λ is the X-ray wavelength (0.154184 nm), β is the full width at half the maximum in radians of the X-ray diffraction peak, and θ is the Bragg angle in degree.

For the highest XRD peak (311) a crystallite size of about 8.9 nm was estimated, which is consistent with the mean size obtained from the TEM analyses. We did not notice significant differences for the full width at half the maximum values at different values of diffraction peaks; thus, we can suppose that the nanoparticles have a spherical shape. This can be also observed in TEM images (Figure 3).

Using Bragg’s law [36], the lattice parameter, a, and interplanar spacing, d, were estimated. The XRD pattern reveals the formation of a magnetite crystalline structure with a lattice parameter about a = 0.8389 nm and d_311_ = 0.253 nm, which are very nearly the values reported for bulk magnetite (a = 0.839 nm and d_311_ = 0.253 nm, respectively) [37]. The presence of sharp and intense XRD peaks confirmed the formation of crystalline nanoparticles with no amorphous phase.

**Figure 7:**
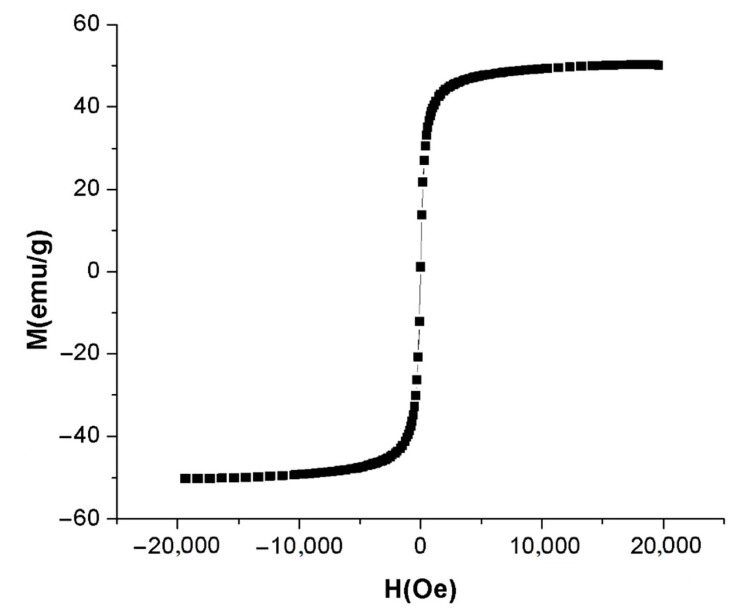
Magnetization curve of the synthesized A-IONPs.

The magnetization curve of the synthesized A-IONPs sample is shown in Figure 7. From VSM experimental data, the saturation value of the specific magnetization of the A-IONP sample was about 50.19 emu/g, while the very low magnetic coercivity was about 3.071 Oe. Thus, we showed the superparamagnetic features of the A-IONPs.

Using magnetization data, the average size of the magnetic core diameter was calculated according to Langevin’s equation [38,39]. The size of large nanoparticles influenced by a low magnetic field can be obtained using the follow equation:

(2) $$d_{M}^{3}=\frac{18\cdot k_{B}\cdot T}{\pi \cdot \mu_{0}\cdot M_{S}\cdot m_{S}}{\left(\frac{\mathrm{dM}}{\mathrm{dH}}\right)}_{H\to 0}$$

where d_M_ is the magnetic nanoparticle diameter, k_B_ is Boltzmann’s constant, T is the absolute temperature of the sample, M_s_ is the saturation magnetization of the sample, and μ_0_ is the permeability of vacuum. Assuming a spherical particle shape, the magnetic nanoparticle size was obtained following the Equation (2) and using the m_s_ value of bulk magnetite (0.48 × 10^6^ A/m) [40].

The graph slope in the origin was determined from the hysteresis plot by linear fitting of the first points neighboring the origin. Thus, the average magnetic diameter of the A-IONP sample was about 8.85 nm at 295 K. This size value is smaller than the nanoparticle size obtained by TEM measurement. Differences between the physical diameter and magnetic diameter values can be assigned to the magnetically “dead” surfactant layer on the magnetic nanoparticles’ surface, which can affect the uniformity or magnitude of magnetization due to quenching of surface moments [41].

**Figure 8:**
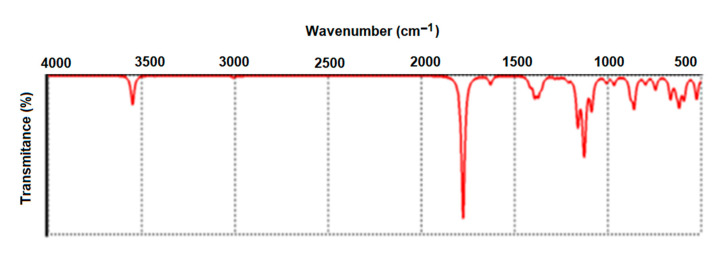
Simulated vibrational spectrum of aspartic acid (Density Functional).

**Figure 9:**
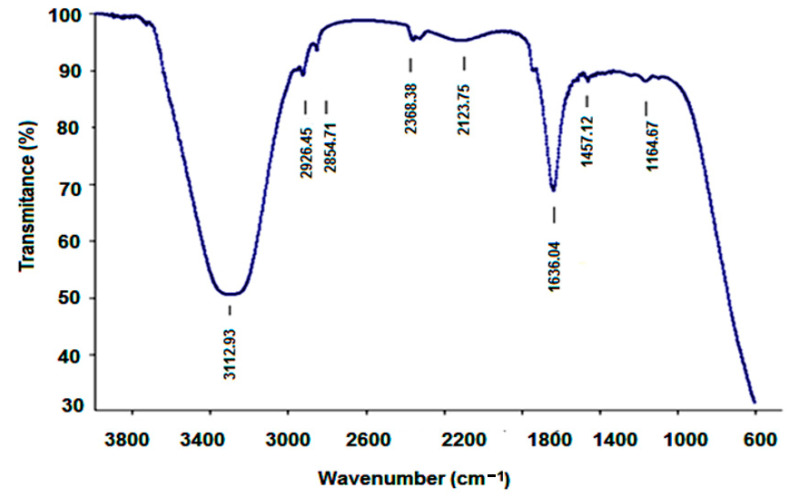
ATR-FTIR pattern of the synthesized A-IONPs.

In Figure 8, the vibrational spectrum is presented as resulting from DFT modeling under Spartan’14. The ATR-FTIR spectrum of A-IONPs aqueous liquid solution is presented in Figure 9 as registered experimentally.

The broad absorption peak at 3312.93 cm^−1^ (Figure 9) can be attributed to stretching vibrations of OH and CH groups, as well as to NH groups that one can find in the theoretical calculated values of 3500−3550 cm^−1^ (Figure 8). The high intensity of this band in the experimental spectrum could suggest residual water molecules in the investigated sample. The weak peaks recorded at 2926.46 cm^−1^ and 2854.71 cm^−1^ suggest the presence of amide groups, and could correspond to the theoretical weak peaks at 2963, 2999, and 3011 cm^−1^.

The 1636.04 cm^−1^ intense band (Figure 9) could be assigned to the C=O stretching vibrations of the aspartic acid molecule attached to the nanoparticle surface through the carboxyl groups; in addition, the water molecule bending may contribute to this band. The experimentally recorded band is different than the theoretical one, which was revealed at 1629 cm^−1^, where the molecule is considered in an isolated state; the interaction with ferrophase cations could be responsible for the band shift in the experimentally recorded spectrum.

Lower wavenumber bands generally correspond to complex vibrations of molecule skeleton, such as those estimated theoretically between 500 and 1400 cm^−1^ and recorded experimentally between 600 and 1457 cm^−1^. At the same time, the ferrophase vibration pattern also overlaps the domain of low wavenumbers; the ATR-FTIR pattern showed a strong peak at around 620 cm^−1^, which corresponds to the stretching vibration of the tetrahedral iron atom—ν (Fe-O) [42] and, according to Cornwell and Schwertmann [43], this IR peak is attributed to magnetite. Based on the results obtained by means of FTIR spectrum, some researchers showed that aspartic acid binds to the surface of the A-IONPs through the side carboxyl group [44]. For some biomedical applications, after aspartic acid is chemisorbed onto the surface of A-IONPs, the grafting of other biomolecules could be accomplished by means of chemical bonds with amine acid.

**Figure 10:**
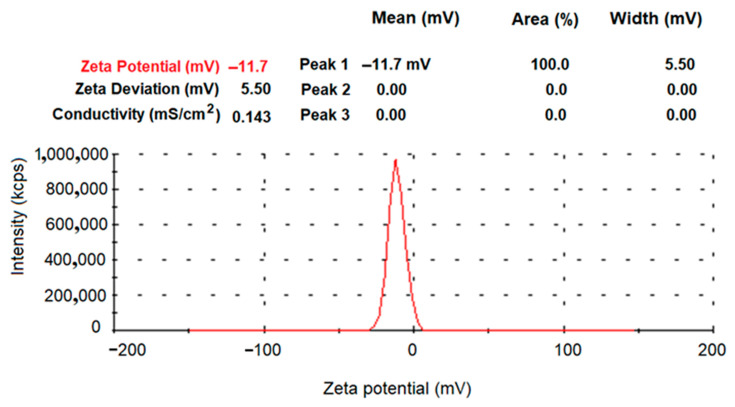
Zeta potential distribution for A-IONPs sample.

Figure 10 shows the zeta potential of A-IONPs. The measurement results showed that the A-IONPs had a zeta potential of about −11.7 mV. Since zeta potential is related to electrostatic repulsion, when steric mechanism also occurs, then suspension stability can be achieved at lower zeta potential—and this seems to be the case with our aspartic acid-coated magnetic nanoparticles [45].

Similar zeta potential values of −10 to −15 mV for pH of around 7 were found in Ostolska et al. (2014) for polyaspartic salt-coated nanoparticles [46]. Nevertheless, the formation of certain large size colloidal aggregates is not excluded, considering the values of the hydrodynamic diameter resulted from NTA data—around 150 nm mean value.

**Figure 11:**
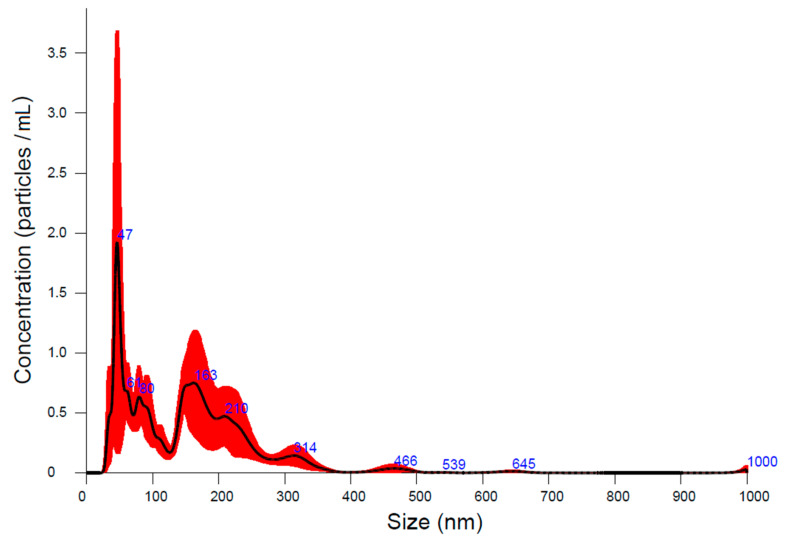
NTA results for A-IONPs dimensional distribution.

Hydrodynamic diameter values revealed by the DLS measurements (Figure 10) suggested that nanoparticles have occasionally agglomerated. Their hydrodynamic size is notably larger than the dimension of their metallic cores measured by TEM analyses; therefore, we could suppose that supplementary steric stabilization of the colloidal suspensions occurs. The suspension investigation by NTA has resulted in a hydrodynamic mean diameter of 153.9 nm (Figure 11), which is similar to the values obtained in other studies [47].

The electric conductivity measured for 10^−3^ volume diluted A-IONP sample was found to be about 11.3 μS/cm at 23.6 °C temperature. For the pH of A-IONPs aqueous solution the 7.2 ± 0.5 value was measured by means of digital WTW Multi 3430 -F meter.

**Figure 12:**
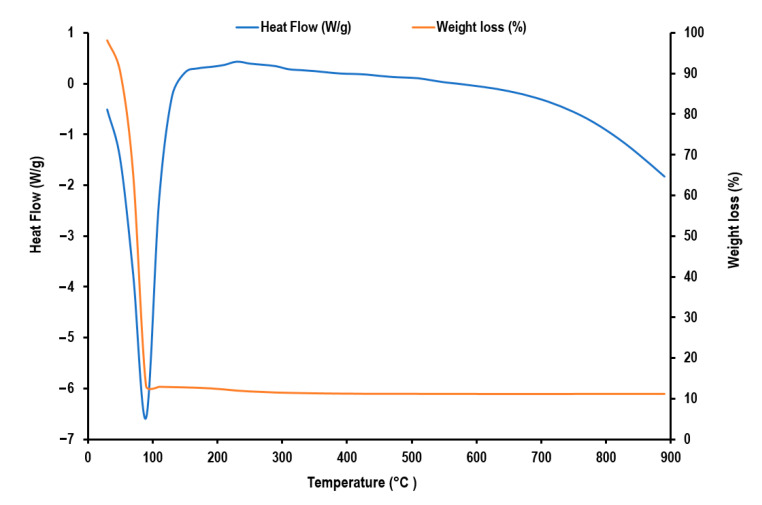
Heat flow (W/g) and weight loss (%) of A-IONPs sample.

Heat flow (W/g) and weight loss (%) of A-IONPs sample are presented in Figure 12. The first decomposition stage in the temperature range from 20 °C to 100 °C represents the solvent evaporation—water residues in our sample, the weight decreasing up to 13.1% from the initial one around of 85 °C. The second important interval for the temperature ranging between 180–265 °C corresponds to the organic breakdown of aspartate chains, the weight decreasing up to 11.2% of the initial one. The total weight losses up to 400 °C were 88.8%, and over 400 °C the weight of sample remained constant. The DSC curve has several exothermic peaks. Of these, the first two correspond to more pronounced weight loss (decreases from 13.1% to 11.2%) which might have occurred because of thermal decomposition of aspartic acid [48].

## 4. Discussion

Compared with other aspartic acid-coated iron oxide nanoparticles prepared by the co-precipitation method [49,50] we obtained a nanoparticle sample characterized by smaller values for the mean physical diameter and similar value of mean crystallite size.

Hydrodynamic diameter seems to have a maximal appearance frequency of about 47 nm (Figure 11). Double modal size distribution could be noticed with secondary maximum at over 150 nm, which most probably correspond to rare large aggregates—the maximum being about three times smaller than the first one, at 47 nm. This is concordant with [50], where individual nanoparticles were reported to have around 37 nm hydrodynamic diameter but larger size aggregates between 100 and 150 nm also were revealed.

For other amino acids like lysine and tyrosine-coating magnetic nanoparticles, prepared by electrochemical deposition, the physical particle mean diameters of 10 nm were estimated together with 8.3 and 8.1 nm crystallite sizes [51]. In the case of proline and tryptophan-stabilized magnetic nanoparticles, physical particle diameters of 7–8 nm and 10–12 nm respectively were evidenced by other authors [15].

The saturation magnetization of around 50 emu/g revealed by our research is concordant with that reported for other magnetic nanoparticles coated with amino acids—around 58 emu/g for L-glutamic acid and about 45 emu/g for L-lysine according to Patel et al. (2009) [14], and around 42 and respectively 52 emu/g for L-lysine and L-arginine according to Ebrahiminezad et al. (2013) [52]. Aspartic acid-coated nanoparticles were found to have about 39 emu/g as reported for aspartic acid iron oxide nanoparticles in Marinescu et al. (2006) [15], of about 47 emu/g according to Singh et al. (2016) [24], and about 35 emu/g in Afradi et al. (2019) [28], for magnetic nanoparticles functionalized with starch and aspartic acid. A very small hysteresis cycle—coercive field of about 3.0711 Oe—and magnetization saturation sustain the superparamagnetic feature of the nanoparticles described in this work.

Zeta potential of about −11.7 mV was found for our magnetic nanoparticle suspension (with about 7.2 pH) compared to −21 mV reported in [49], while for arginine, lysine, and polylysine-coated magnetic nanoparticles Zeta potentials between −11 mV and −20 mV were measured at pH values of 7 and 8.5, respectively.

IR spectrum has shown the binding of aspartic acid molecule to the ferrophase surface; the main vibration bands being shifted in the wavenumber scale when compared to isolated aspartic acid molecule.

These results contribute towards improving the iron oxide nanoparticle synthesis research field. Due to its properties, this iron oxide nanoparticle sample is suitable for biomedical applications. 

## 5. Conclusions

We succeeded in preparing A-IONP colloidal solutions, suitable for biomedical applications due to dominant small particles, anarrow size distribution, mean physical diameter of about 9.17 ± 2.2 nm, and good stability in spite of rare large aggregates suggested by the hydrodynamic diameter. The EDS analysis on the elemental compositions of nanoparticle sample denoted the presence of iron and oxygen. The XRD analysis of nanoparticles sample revealed the magnetite crystallinity properties. Average crystallite size was about 8.98 nm, obtained from XRD pattern using Scherrer equation. The ATR-FTIR spectrum showed that aspartic acid was chemisorbed onto the surface of A-IONPs. Our biocompatible iron oxide nanoparticle sample was synthesized by a fast and cheap co-precipitation method at low cost. In this synthesis method, aspartic acid plays an important role as a surface-modifying agent, as an amino acid with a relevant biological role. All results recommend aspartic acid for the coating of the magnetic nanoparticle surface, which ensures the dispersion in the aqueous environment—as required by biomedical applications. Further experiments will be developed with a focus on the influence of this type of nanoparticles on plant tissues, to investigate its genotoxic potential and phytotoxicity. A similar sample of A-IONPs, synthesized using another protocol, has already been used in a biological study to evaluate its genotoxic influence on the root tip cells of Zea mays seeds. In that case, the mitotic division of cells was stimulated under A-IONP influence with a low rate of aberrant cell occurrence [53].

## Data Availability

Not applicable.

## References

[1] Li T., Sun M., Wu S. (2022). State-of-the-Art review of electrospun gelatin-based nanofiber dressings for wound healing applications. Nanomaterials.

[2] Lai W.F., Gui D., Wong M., Döring A., Rogach A.L., He T., Wong W.T. (2021). A self-indicating cellulose-based gel with tunable performance for bioactive agent delivery. J. Drug Deliv. Sci. Technol..

[3] Yan J., Yao Y., Yan S., Gao R., Lu W., He W. (2020). Chiral protein supraparticles for tumor suppression and synergistic immunotherapy: An enabling strategy for bioactive supramolecular chirality construction. Nano Lett..

[4] Salarpour S., Barani M., Pardakhty A., Khatami M., Chauhan N.P.S. (2022). The application of exosomes and exosome-nanoparticle in treating brain disorders. J. Mol. Liq..

[5] Gupta A.K., Gupta M. (2005). Synthesis and surface engineering of iron oxide nanoparticles for biomedical applications. Biomaterials.

[6] Gu H., Xu K., Xu C., Xu B. (2006). Biofunctional magnetic nanoparticles for protein separation and pathogen detection. Chem. Commun..

[7] Mashhadi Malekzadeh A., Ramazani A., Tabatabaei Rezaei S.J., Niknejad H. (2017). Design and construction of multifunctional hyperbranched polymers coated magnetite nanoparticles for both targeting magnetic resonance imaging and cancer therapy. J. Colloid Interface Sci..

[8] Li L., Gao F., Jiang W., Wu X., Cai Y., Tang J., Gao X., Gao F. (2016). Folic acid-conjugated superparamagnetic iron oxide nanoparticles for tumor-targeting MR imaging. Drug Deliv..

[9] Hafeli U., Schutt W., Teller J., Zborowski M. (1997). Scientific and Clinical Applications of Magnetic Carriers.

[10] Demirer G.S., Okur A.C., Kizilel S. (2015). Synthesis and design of biologically inspired biocompatible iron oxide nanoparticles for biomedical applications. J. Mater. Chem. B.

[11] Arias L.S., Pessan J.P., Vieira A., Lima T., Delbem A., Monteiro D.R. (2018). Iron Oxide Nanoparticles for Biomedical Applications: A Perspective on Synthesis, Drugs, Antimicrobial Activity, and Toxicity. Antibiotics.

[12] Qu H., Ma H., Zhou W., O’Connor C. (2012). In situ surface functionalization of magnetic nanoparticles with hydrophilic natural amino acids. Inorg. Chim. Acta.

[13] Berry C. (2005). Possible exploitation of magnetic nanoparticle-cell interaction for biomedical applications. J. Mater. Chem..

[14] Patel D., Chang Y., Lee G.H. (2009). Amino Acid Functionalized Magnetite Nanoparticles in Saline Solution. Curr. Appl. Phys..

[15] Marinescu G., Patron L., Culita D.C., Neagoe C., Lepadatu C.I., Balint I., Bessais L., Cizmas C.B. (2006). Synthesis of Magnetite Nanoparticles in the Presence of Aminoacids. J. Nanopart. Res..

[16] Sousa M.H., Rubim J.C., Sobrinho P.G., Tourinho F.A. (2001). Biocompatible magnetic fluid precursors based on aspartic and glutamic acid modified maghemite nanostructures. J. Magn. Magn. Mater..

[17] Durmus Z., Kavas H., Toprak M.S., Baykal A., Altınçekiç T.G., Aslan A., Bozkurt A., Coşgun S. (2009). L-lysine coated iron oxide nanoparticles: Synthesis, structural and conductivity characterization. J. Alloys Compd..

[18] Park J.Y., Choi E.S., Baek M.J., Lee G.H. (2009). Colloidal stability of amino acid coated magnetite nanoparticles in physiological fluid. Mater. Lett..

[19] Berovic M., Berlot M., Kralj S., Makovec D. (2014). A New Method for the Rapid Separation of Magnetized Yeast in Sparkling Wine. Biochem. Eng. J..

[20] Ebrahiminezhad A., Varma V., Yang S., Ghasemi Y., Berenjian A. (2016). Synthesis and Application of Amine Functionalized Iron Oxide Nanoparticles on Menaquinone-7 Fermentation: A Step towards Process Intensification. Nanomaterials.

[21] Jin Y., Liu F., Shan C., Tong M., Hou Y. (2014). Efficient bacterial capture with amino acid modified magnetic nanoparticles. Water Res..

[22] Rhee J.E., Sheng W., Morgan L.K., Nolet R., Liao X., Kenney L.J. (2008). Amino acids important for DNA recognition by the response regulator OmpR. J. Biol. Chem..

[23] Dalangin R., Kim A., Campbell R.E. (2020). The Role of Amino Acids in Neurotransmission and Fluorescent Tools for Their Detection. Int. J. Mol. Sci..

[24] Singh D., Singh S.K., Atar N., Krishna V. (2016). Amino acid functionalized magnetic nanoparticles for removal of Ni(II) from aqueous solution. J. Taiwan Inst. Chem. Eng..

[25] Ebrahiminezhad A., Ghasemi Y., Rasoul-Amini S., Barar J., Davaran S. (2012). Impact of amino-acid coating on the synthesis and characteristics of iron-oxide nanoparticles (IONs). Bull. Korean Chem. Soc..

[26] Ebrahiminezhad A., Davaran S., Rasoul-Amini S., Barar J., Moghadam M., Ghasemi Y. (2012). Synthesis, characterization and anti-Listeria monocytogenes effect of amino acid coated magnetite nanoparticles. Curr. Nanosci..

[27] Nosrati H., Salehiabar M., Davaran S., Ramazani A., Manjili H.K., Danafar H. (2017). New advances strategies for surface functionalization of iron oxide magnetic nano particles (IONPs). Res. Chem. Intermed..

[28] Afradi N., Foroughifar N., Pasdar H., Qomi M. (2019). Aspartic-acid-loaded starch-functionalized Mn–Fe–Ca ferrite magnetic nanoparticles as novel green heterogeneous nanomagnetic catalyst for solvent-free synthesis of dihydropyrimidine derivatives as potent antibacterial agents. Res. Chem. Intermed..

[29] Berger P., Adelman N.B., Beckman K.J., Campbell D.J., Ellis A.B., Lisensky G.C. (1999). Preparation and properties of an aqueous ferrofluid. J. Chem. Educ..

[30] Răcuciu M. (2011). Biocompatible Magnetic Nanofluids: Synthesis, Characterisation and Applications.

[31] Hehre W.J., Radom L., Schleyer P.V.R., Pople J.A. (1986). Ab Initio Molecular Orbital Theory.

[32] Young D. (2001). Computational Chemistry.

[33] Movre Šapić I., Vidak A., Dananić V. (2019). Experimental and theoretical study of aspartic acid. Bull. Chem. Technol. Bosnia Herzeg..

[34] Stefaniu A., Savoiu V.G., Lupescu I., Iulian O. (2016). Computational study on 3D structure of L-aspartic acid and L-glutamic acid: Molecular descriptors and properties. Ovidius Univ. Ann. Chem..

[35] Patterson A. (1939). The Scherrer formula for X-Ray particle size determination. Phys. Rev..

[36] Cullity B.D., Stock S.R. (2001). Elements of X-ray Diffraction.

[37] Blaney L. (2007). Magnetite (Fe_3_O_4_): Properties, Synthesis, and Applications. Lehigh Rev. Lehigh Preserv..

[38] Massart R. (1981). Preparation of aqueous magnetic liquids in alkaline and acidic media. IEEE Trans. Magn..

[39] Kákay A., Gutowski M.W., Takacs L., Franco V., Varga L.K. (2004). Langevin granulometry of the particle size distribution. J. Phys. A Math. Gen..

[40] Rosensweig R.E. (1985). Ferrohydrodynamics.

[41] Kaiser R., Miskolczy G. (1970). Magnetic properties of stable dispersions of subdomain magnetite particles. J. Appl. Phys..

[42] Predoi D. (2007). A study on iron oxide nanoparticles coated with dextrin obtained by coprecipitation. Dig. J. Nanomater. Biostruct..

[43] Cornwell R.M., Schwertmann U. (2003). The Iron Oxides: Structure, Properties, Reactions, Occurrences and Uses.

[44] Mikhaylova M., Kim D.K., Berry C.C., Zagorodni A., Toprak M., Curtis A.S.G., Muhammed M. (2004). BSA immobilization on amine-functionalized superparamagnetic iron oxide nanoparticles. Chem. Mater..

[45] Lunardi C.N., Gomes A.J., Rocha F.S., de Tommaso J., Patience G.S. (2021). Experimental methods in chemical engineering: Zeta potential. Can. J. Chem. Eng..

[46] Ostolska I., Wiśniewska M. (2014). Application of the zeta potential measurements to explanation of colloidal Cr2O3 stability mechanism in the presence of the ionic polyamino acids. Colloid Polym. Sci..

[47] Nădejde C., Puscașu E., Brinza F., Ursu L., Creangă D., Stan C. (2015). Preparation of soft magnetic materials and characterization with investigation methods for fluid samples. U. Politech. Buch. Ser. A.

[48] Weiss I.M., Muth C., Drumm R., Kirchner H.O.K. (2018). Thermal decomposition of the amino acids glycine, cysteine, aspartic acid, asparagine, glutamic acid, glutamine, arginine and histidine. BMC Biophys..

[49] Salehiabar M., Nosrati H., Davaran S., Danafar H., Manjili H.K. (2018). Facile synthesis and characterization of L-Aspartic acid coated iron oxide magnetic nanoparticles (IONPs) for biomedical ap-plications. Drug Res..

[50] Pušnik K., Peterlin M., Cigić I.K., Marolt G., Kogej K., Mertelj A., Gyergyek S., Makovec D. (2016). Adsorption of amino acids, aspartic acid, and lysine onto iron-oxide nanoparticles. J. Phys. Chem. C.

[51] Karimzadeh I., Aghazadeh M., Doroudi T., Ganjali M.R., Kolivand P.H., Gharailou D. (2016). Amino Acid Coated Superparamagnetic Iron Oxide Nanoparticles for Biomedical Applications Through a Novel Efficient Preparation Method. J. Clust. Sci..

[52] Ebrahiminezhad A., Ghasemi Y., Rasoul-Amini S., Barar J., Davaran S. (2013). Preparation of novel magnetic fluorescent nanoparticles using amino acids. Colloids Surf. B Biointerfaces.

[53] Răcuciu M. (2020). Iron oxide nanoparticles coated with aspartic acid and their genotoxic impact on root cells of *Zea mays* embryos. Rom. Rep. Phys..

